# Performance evaluation of a dedicated computed tomography scanner used for virtual simulation using in-house fabricated CT phantoms

**DOI:** 10.4103/0971-6203.25667

**Published:** 2006

**Authors:** D. S. Sharma, S. D. Sharma, K. K. Sanu, S. Saju, D. D. Deshpande, S. Kannan

**Affiliations:** Department of Medical Physics, Tata Memorial Hospital, Parel, Mumbai, India; * Department of Radiological Physics and Advisory Division, Bhabha Atomic Research Center, Anushaktinagar, Mumbai, India

**Keywords:** CTDI, CT-simulation, image quality, quality assurance, virtual simulation

## Abstract

Comprehensive tests on single slice CT scanner was carried out using in-house fabricated phantoms/test tools following AAPM recommended methods to independently validate the auto-performance test (APT) results. Test results of all the electromechanical parameters were found within the specified limits. Radiation and sensitivity profile widths were within ± 0.05 cm of the set slice thickness. Effective energy corresponding to nominal kVp of 80, 110 and 130 were 49.99, 55.08 and 59.48 keV, respectively. Percentage noise obtained by APT was 1.32% while the independently measured value was 0.38%. Observed contrast resolutions by independent method at 0.78% and 12% contrast difference were 4 mm and 1.25 mm (= 4 lp/cm) respectively. However, high contrast resolution (limiting spatial resolution) by APT at 50, 10 and 2% MTF levels were 9, 12.5 and 14.1 lp/cm respectively. Difference in calculated and measured CT numbers of water, air, teflon, acrylic, polystyrene and polypropylene were in the range of 0 to 24 HU, while this difference was 46 and 94 HU in case of nylon and bakelite respectively. The contrast scale determined using CT linearity phantom was 1.998×10^−4^ cm^−1^/CT number. CT dose index (CTDI) and weighted CTDI (CTDI_w_) measured at different kVp for standard head and body phantoms were smaller than manufacturer-specified and system-calculated values and were found within the manufacturer-specified limit of ± 20%. Measured CTDIs on surface (head: 3.6 cGy and body: 2.6 cGy) and at the center (3.3 cGy, head; and 1.2 cGy, body) were comparable to reported values of other similar CT scanners and were also within the industry-quoted CTDI range. Comprehensive QA and independent validation of APT results are necessary to obtain baseline data for CT virtual simulation.

## Introduction

Computed tomography virtual simulation (CT-VSIM) is the simulation performed on virtual patient created from the volumetric patient scan using software-created virtual simulator. It is a three-dimensional simulation and verification procedure, which has been shown to offer clear advantages over physical simulation in reducing field size[1] and simulation time.[2,3] In physical simulation, the accuracy and reproducibility of simulation data depends mainly on mechanical performance of the radiotherapy simulator, while the accuracy and robustness of CT-VSIM procedure depends mainly on the performance of the CT scanner.[5] However, set up errors of CT-VSIM and physical simulation procedures are comparable.[1,4] Performance of the CT scanner is generally tested following manufacturer recommended procedures during installation and periodically thereafter, using manufacturer supplied phantoms. Auto-performance test (APT) software that controls the scanning parameter settings and generates the test results automatically as per predefined protocol is also available with modern CT scanners. Moreover, APT provides CT performance results of the image quality related and a few mechanical parameters only and does not include CT performance related to radiation safety. American Association of Physicists in Medicine (AAPM) Radiation Therapy Committee Task Group 66 recommends[5] a comprehensive test of the CT scanner and independent validation of baseline parameters before its use for CT-VSIM. Independent validation of the APT software is also necessary before its use for routine quality assurance (QA). In light of the recent recommendations, comprehensive tests were carried out on a dedicated CT scanner used for virtual simulation procedure at our hospital using in-house fabricated CT phantoms to obtain the baseline data and validate the test results of APT software.

## Materials and Methods

CT-VSIM procedure is realized at our center using a dedicated 70 cm aperture CT scanner (Somatom Emotion, Siemens Medical Systems, Germany), a SmartSim 6.2 virtual simulation (VSIM) workstation (ADAC Pinnacle, Philips Medical Systems, USA) and a moving laser system (Gammex RMI, Widdleton, USA). Auto-performance tests were carried out using manufacturer supplied test phantoms (alignment phantom, MTF phantom, water phantom and slice thickness phantom) and inbuilt APT software following manufacturer recommended procedures. The test results obtained during APT were: congruence of gantry laser and imaging plane center, accuracy of table longitudinal movement, slice thickness accuracy, CT number uniformity, noise and modulation transfer function (MTF). The set of data thus obtained was validated independently and are used as baseline data set. Independent verification of baseline data and other QA tests recommended by AAPM[5,6] were carried out using in-house fabricated CT phantoms and test tools. Independent tests were carried out to evaluate the electromechanical, image quality related and radiation safety related performance of this CT scanner. Details about the in-house fabricated phantoms/test tools and their reliability for use in QA of CT scanners is available elsewhere.[8,9]

### Electromechanical tests

The CT performance parameters evaluated under electromechanical category were: congruence of gantry laser and imaging plane, localization of CT and pseudo CT center, orthogonality of table top long axis to imaging plane, accuracy of table vertical and longitudinal movement, gantry tilt accuracy, radiation and sensitivity profile widths and tests on X-ray generator.

Congruence of gantry laser with center of imaging plane and gantry tilt accuracy were verified using ready pack film (Kodak X-V) following the methods described in AAPM Report[6] 39. CT center and pseudo CT center (an arbitrary point located at 60 cm inferior to CT center) were localized using a commercially available laser calibration phantom (Gammex RMI, Widdleton, USA). For this purpose, transverse images of the mid-plane of two parallel slabs (having a separation of 60 cm) of the laser calibration phantom were acquired using 0.1 cm slice width. This setup was also used to verify the accuracy of longitudinal table motion and orthogonality of table top longitudinal axis to the image acquisition plane. Calibrations of table linear scales were verified by moving the table both vertically and longitudinally in steps of 1 cm using a calibrated measuring scale. Table indexing accuracy and reproducibility were tested by irradiating ready pack films placed perpendicular to scan plane. The irradiation of the film was carried out using 0.1 cm slice width and scanner-controlled longitudinal positions of the table at 0, 2, 5, 10 and 20 cm.

**Figure 1:**
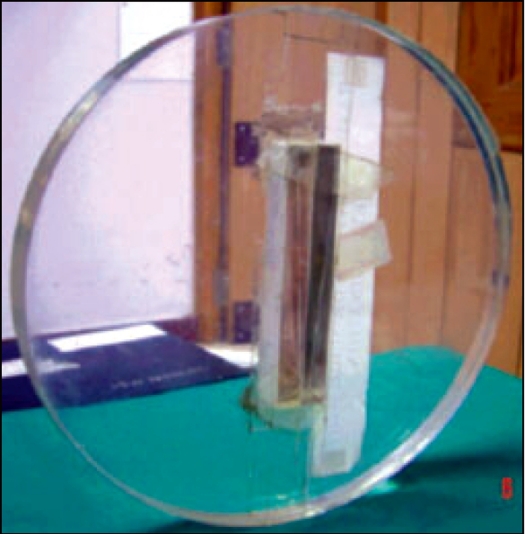
In-house fabricated sensitivity profile phantom

*Radiation and sensitivity profile widths:* A ready pack film placed horizontal to the table top at the CT center was exposed using available slice thicknesses of 0.1, 0.2, 0.3, 0.5, 0.8 and 1.0 cm respectively. Exposed films were scanned using VXR-16 film scanner (Vidar Systems Corporation, USA) with 0.01 cm step size and 285 pixel resolution. OmniPro Accept 6.2 analyzing software (Scanditronix Welhofer, Sweden) was used to measure the full width at half maximum (FWHM) of optical density profiles of the images so obtained. These FWHM values represent the radiation profile widths. Independent verification of the sensitivity profile was performed using an in-house fabricated slice width phantom shown in Figure 1. It contains two sets of copper strips angled at 23° that are used to determine slice thickness. The phantom was scanned in helical mode using different slice thickness. The resulting images were transferred to ADAC virtual simulation workstation and CT number profiles were generated along the long axis of the copper strips and sensitivity profile width was calculated using the relation

(1) $${(\text{Sensitivity profile width)}}_{\text{i}}={\left(\text{FWHM}\right)}_{\text{i}}\times \tan\text{ 23}{}^{\circ}\;\mathrm{(1)}$$

Where (FWHM)_i_ is the average FWHM value of CT number profile taken along the vertical line of the copper strips corresponding to slice thickness i.

*X-ray generator:* Tests on the X-ray generator include evaluation of peak potential (kVp), timer accuracy (s), mAs linearity and reproducibility and determination of effective energy by measuring half value thickness (HVT). Non-invasive measurement of kVp for different mAs was performed using Mult-O-Meter 600 (Unfors Instruments, Sweden) following the method described in AAPM[6] Report 39. Linearity of mAs for different kVp was verified by obtaining the product of dose and time at different mA and timer settings. HVT in copper at 80, 110 and 130 kVp were measured using a pencil ionization chamber (Unfors Instruments, Sweden) positioned at the center of an in-house fabricated HVT phantom. The HVT phantom is a 20 cm diameter, 2.5 cm wide and 0.5 cm thick lucite ring on which copper strips of width 2.5 cm and different thicknesses can be rolled over. HVT values so determined were subsequently used to obtain the corresponding effective energy and hence linear attenuation coefficient for theoretical calculation of CT number for different materials.

### Image quality related tests

*Image uniformity and pixel noise:* Image of 20 cm diameter and 2.5 cm thick in-house fabricated water phantom was obtained using the scanner settings, which was used during APT. Mean CT number of water contained within a circular region of 1 cm^2^ (about 400 pixels) was then obtained at different locations corresponding to 12, 3, 6 and 9 o'clock positions and at the center of the phantom using system software. Image homogeneity defined as the edge-to-center difference in mean CT number was then calculated. Image noise was determined using the relation

(2) $$\%\text{ of noise}=(\sigma \times \text{CS}\times 100)/{\text{μ}}_{\text{w}}\;\mathrm{(2)}$$

Where σ = standard deviation of CT numbers of water within the region of interest; CS is the contrast scale defined as CS = [(μ_m_ - μ_w_)/(CT_m_ - CT_w_)], where μ_m_ and μ_w_ are the linear attenuation coefficients for the subject material and water respectively and CT_m_ and CT_w_ are the measured CT numbers of the subject material and water. CS was determined using CT number linearity phantom, which is described later.

**Figure 2:**
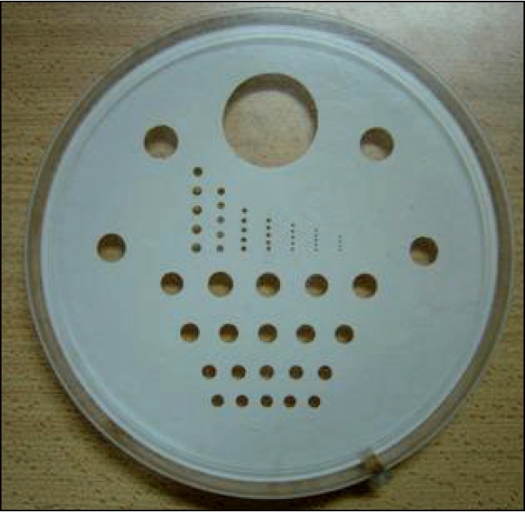
In-house fabricated low-contrast resolution phantom

*Low-contrast resolution:* Low-contrast resolution measures the ability of CT scanner to distinguish relatively large objects that differ only slightly in density from background and it was determined using the in-house fabricated low-contrast phantom shown in Figure 2. This phantom is based on the principle of partial volume effect and contains a 0.05 cm thick polystyrene circular sheet on which hole patterns of various diameters are drilled. This polystyrene sheet is placed at the central plane of a 20 cm diameter circular phantom filled with 10% dextrose solution. The percentage contrast difference between polystyrene-plus-dextrose and dextrose was determined from the transverse image of the phantom taken using slice width of 1.0 cm at 110 kVp.

**Figure 3:**
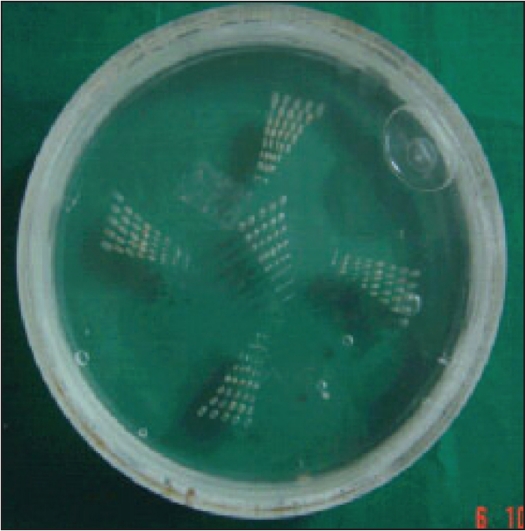
In-house fabricated high-contrast resolution phantom

*High-contrast resolution:* High contrast resolution (limiting spatial resolution) is defined as the minimum resolvable diameter of an object embedded in a uniform medium that differs in density from its background and it was determined using the in-house fabricated high-contrast phantom shown in Figure 3. It consists of 20 cm diameter and 2.5 cm thick acrylic disc with five sets (one at the center and four at periphery 90° apart) of hole-pair patterns drilled on it. The distance between the centers of holes is twice the diameter of the hole. The high contrast is obtained between acrylic and water (CT number difference of 120 ~ 12% contrast difference). The smallest resolvable hole-pair pattern was visualized from the image of this phantom and high-contrast resolution was determined in terms of lp/cm. [Figure 3]

**Figure 4:**
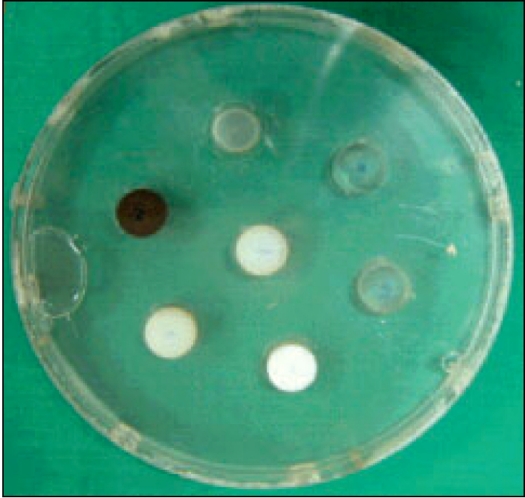
In-house fabricated CT number linearity phantom

*CT number linearity:* The CT number linearity phantom [Figure 4] contains seven cylindrical inserts of different materials (air, Perspex, polypropylene, bakelite, polystyrene, nylon and teflon) that simulate attenuation coefficient of various organs ranging from lung to bone. This phantom was scanned using 0.8 cm slice thickness at 130 kVp. CT numbers for these materials were determined from their respective images with the help of system software. The CT numbers of these materials were calculated using the respective linear attenuation coefficient taken from Hubbell and Seltzer[10] (for 60 keV) and were compared with the directly measured values.

### Radiation safety

CT dose index (CTDI), which is a measure of the patient dose, was measured using in-house fabricated standard cylindrical head (16 cm diameter and 15 cm long) and body (32 cm diameter and 15 cm long) phantoms and the CT pencil ionization chamber (Mult-O-Meter 600, Unfors Instruments, Sweden). These two phantoms contain five longitudinal holes (one at the center and four at the periphery corresponding to 12, 3, 6 and 9 o'clock positions) suitable to insert the CT pencil ionization chamber. CTDI values on the surface and at the center were determined by inserting the CT pencil ionization chamber in the peripheral holes and at the central hole of these phantoms respectively. The weighted CTDI (CTDI_w_) was then calculated using the expression

(3) $${\text{CTDI}}_{\text{w}}=2/3\text{ CTDI }(\text{surface})+1/3\text{ CTDI }(\text{center})\;\mathrm{(3)}$$

Where CTDI (surface) is the average of CTDI values measured at the four peripheral positions and CTDI (center) is the CTDI value measured at the center of the phantom. All measurements were carried out using 1.0 cm slice thickness in sequential (axial) mode. CTDI values were determined for nominal X-ray energies in the range of 80 - 130 kVp at 100 mAs, while CTDI_w_ was measured for commonly used scan protocols of head and pelvis. Corresponding values of CTDI and CTDI_w_ displayed on the console were recoded for comparison.

## Results

### Electromechanical tests

**Figure 5:**
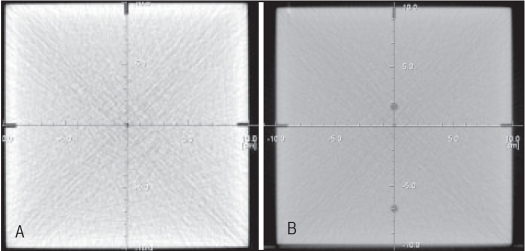
Transverse images of the two faces of laser calibration phantom representing (A) CT center (Z = 0) and (B) Pseudo CT center (Z = 60 cm)

**Figure 6:**
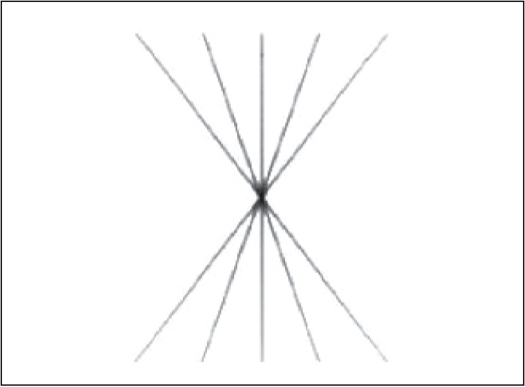
Strip field of 0.1 cm exposed on the ready pack film from different angular positions of the gantry

Coincidence between laser and imaging plane was within 0.05 cm both in APT and in independent methods using ready pack film and laser calibration phantom. [Figure 5] shows 0.1 cm thick transverse images taken at the mid-plane of two parallel slabs of laser calibration phantom separated by 60 cm. CT center (X = 0, Y = 0 and Z = 0) and pseudo CT center (X = 0, Y = 0 and Z = 60 cm) were found localized within ± 0.05 cm shift along Y (vertical) direction. Table movement was accurate to within ± 0.05 cm for table motion of 60 cm in cranio-caudal (Z) direction. Exactly same value of the lateral coordinate (X = 0) in case of both CT and pseudo CT centers demonstrates the exact orthogonality of table top long axis with the imaging plane. Calibrations of mechanical and digital scales of CT table were found accurate to within ± 0.2 cm. Table indexing under scanner control were found accurate and reproducible within a maximum deviation of 0.1 cm both during APT and during independent validation. Figure 6 represents image of the 0.1 cm strip field exposed from different angular positions of the gantry. From this image, it was determined that the gantry tilt was accurate to within ± 0.5°.

*Radiation and sensitivity profile widths:* Values of the set slice thickness and measured radiation and sensitivity profile widths are given in Table 1. The measured radiation profile widths are in good agreement to the corresponding set slice thickness and are within the manufacturer specified limit of 0.05 cm. Sensitivity profile widths measured using in-house fabricated phantom are also in good agreement to both the set slice widths and sensitivity profile widths measured using manufacturer supplied phantom during APT. Maximum difference of 0.05 cm is observed between 0.1 cm set slice width and its sensitivity profile width measured using in-house fabricated phantom.

**Table 1:** Radiation and sensitivity profile widths measured by independent method using in-house phantom and by auto-performance test using manufacturer supplied phantom and the software

*Set slice thickness (cm)*	*Radiation profile width (cm)*	*Sensitivity profile width (cm)*	
		
	*Independent method*	*APT*	*Independent method*
0.10	0.13	0.13	0.15
0.20	0.20	0.22	0.21
0.30	0.29	0.30	0.32
0.50	0.50	0.51	0.47
0.80	0.79	0.81	0.78
1.00	1.00	0.96	1.02

*X-ray generator:* Set and measured kVp for different mAs was found to agree within the prescribed limit[5,6] of ± 2 kVp. Coefficient of mAs linearity was within the acceptable criteria of <0.1. The measured half value thickness in copper for nominal X-ray energies of 80, 110 and 130 kVp are 0.029, 0.037 and 0.047 cm respectively. The linear attenuation coefficient (μ) for copper derived from these measured HVTs are 0.237, 0.186 and 0.146 cm^−1^ respectively. Accordingly, effective energies[10] of these X-rays are 49.99, 55.08 and 59.48 keV respectively. The linear attenuation coefficient of different materials in the CT number linearity phantom was taken for 60 keV (130 MV) from the data book of Hubbell and Seltzer[10] to calculate the CT number of these materials.

### Image quality related tests

*Image uniformity and pixel noise:* As is evident from Table 2, the uniformity of mean CT number of water at 110 and 130 kVp is less than 1 HU both in case of APT and in independent measurement. Table 3 shows the standard deviation of the CT number of water and image noise measured for head phantom during APT and during independent measurement. Manufacturer (or APT) specifies image noise as standard deviation of CT numbers of water at the center of the uniform water phantom. For head type phantom, it is 13.58 HU at 110 kVp and 11.55 HU at 130 kVp. Percent image noise (calculated using equation 2) corresponding to these values of standard deviations are 1.32% at 110 kVp and 1.12% at 130 kVp. However, the noise level is 0.38 and 0.33% when calculated using independently measured standard deviations (average values) of 3.96 and 3.41 HU at 110 and 130 kVp respectively. It is important to mention here that μ_w_ of 0.207 cm^−1^ was used while calculating the percent noise.

**Table 2:** CT number uniformity of water measured at different kVp (head technique)

*Location*	*Mean CT number of water*			
	
	*110 kVp*		*130 kVp*	
		
	*APT*	*In-house phantom*	*APT*	*In-house phantom*
Center	1.36	0.4	0.01	0.15
12 o' clock	1.41	0.4	0.24	0.15
3 o' clock	1.24	0.8	0.29	0.30
6 o' clock	1.04	0.2	0.22	0.60
9 o' clock	1.68	0.2	0.14	0.15
Uniformity	0.02	0	0.21	0.15

**Table 3:** Standard deviation and image noise (head-type phantom and head technique)

*Location*	*Standard deviation of CT number of water*			
	
	*110 kVp*		*130 kVp*	
		
	*APT*	*In-house phantom*	*APT*	*In-house phantom*
Center	13.58	4.10	11.55	3.58
12 o' clock	-	3.89	-	3.44
3 o' clock	-	3.91	-	3.30
6 o' clock	-	3.93	-	3.46
9 o' clock	-	3.95	-	3.26
Noise %	1.32	0.38	1.12	0.33

*Low- and high-contrast resolution:* Smallest visible hole was recorded by careful visual inspection of the image of low-contrast resolution phantom. It was then calculated that the low-contrast resolution is 0.4 cm at 0.78% contrast difference. The smallest visible hole pattern on the image of high-contrast resolution phantom was carefully recorded and average high-contrast (spatial) resolution was found as 4 lp/cm at a contrast difference of 12%. APT-rendered MTF at 50, 10 and 2% contrast difference are 9, 12.5 and 14.1 lp/cm respectively.

**Figure 7:**
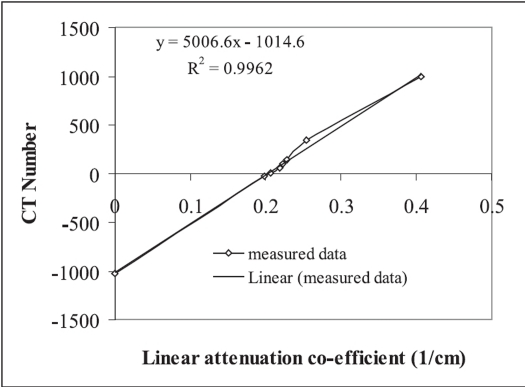
CT number linearity and contrast scale

*CT number accuracy and linearity:* Table 4 shows the comparison of nominal, theoretically calculated and experimentally measured CT number of different materials for an effective energy of 60 keV (experimentally measured effective energy corresponding to 130 kVp). Measured CT numbers are higher than both nominal and theoretically calculated values for all the materials of the CT linearity phantom. Measured CT numbers of water and air agree with the manufacturer specified values of 0 and −1024 HU. CT number difference in the range of 6 - 22 HU are observed for polypropylene, acrylic and teflon when measured values are compared with nominal and calculated values. The difference is even more for bakelite and nylon and reaches up to 94 HU and 46 HU respectively. The linear attenuation coefficient of different materials are plotted against measured CT number and are shown in Figure 7. Linear regression analysis of the data gives contrast scale (CS) of [1.998×10^−4^ cm^−1^/CT number] with a good correlation coefficient of 0.9963.

**Table 4:** Nominal, calculated and measured CT number for different materials representing lung to bone

*Material*	*Nominal[11] CT number*	*Calculated CT number*	*Measured CT number*
Air	−1000	−1000	−1024
Water	0	0	0
Acrylic	120	120	140
Teflon	990	970	992
Polystyrene	--	−43	−26
Bakelite	--	243	337
Polypropylene	90	94	100
Nylon	--	59	105

### Radiation safety related tests

*CT dose index:* Table 5 shows the CTDI_w_ and Tables 6 and 7 list CTDI of head and body phantoms for the most commonly used tube voltages of 80, 110 and 130 kVp. Measured and system calculated CTDI_w_ values agree within the manufacturer specified limit of ±20%. Measured values are smaller than the system calculated values and the maximum difference between these two data are −16.18% for head and −17.62% for body phantoms. Similar difference between the measured and manufacturer quoted CTDI values was also observed for all X-ray potentials for which CTDI was measured. The difference between the independently measured and system-displayed values of CTDI (center) is −10% for head phantom and 17.67% for body phantom, while the difference between the independently measured and system-displayed values of CTDI (surface) is −7.41% for head phantom and −10.67% for body phantom.

**Table 5:** Experimentally measured and system-calculated values (in cGy) of weighted computed tomography dose index (CTDI_w_) for head and body phantoms/protocols

*Head phantom/protocol*				*Body phantom/protocol*			
	
*kV/mAs*	*Measured*	*System calculated*	*% diff.*	*kV/mAs*	*Measured*	*System calculated*	*% diff.*
80/260	1.41	1.69	−16.18	80/225	0.52	0.63	−17.62
110/195	2.54	2.98	−15.05	110/225	1.26	1.51	−16.12
130/225	4.42	5.13	−13.94	130/225	2.03	2.43	−16.63

**Table 6:** Experimentally measured and manufacturer's quoted values (in cGy) of computed tomography dose index (CTDI) for head phantom

*kV/mAs*	*Surface*			*Center*		
		
	*Measured*	*Quoted*	*% diff.*	*Measured*	*Quoted*	*% diff.*
80/100	0.57	0.61	− 7.41	0.49	0.55	− 10.0
110/100	1.33	1.41	− 5.53	1.23	1.35	− 8.32
130/100	2.02	2.09	− 3.26	1.85	2.01	− 7.86

**Table 7:** Experimentally measured and manufacturer's quoted values (in cGy) of computed tomography dose index (CTDI) for body phantom

*kV/mAs*	*Surface*			*Center*		
		
	*Measured*	*Quoted*	*% diff.*	*Measured*	*Quoted*	*% diff.*
80/100	0.29	0.31	− 5.57	0.12	0.14	− 16.55
110/100	0.68	0.76	− 10.67	0.33	0.40	− 17.50
130/100	1.09	1.15	− 5.24	0.53	0.65	− 17.67

## Discussion

At the time of installation of our new CT scanner for virtual simulation, the specified performance characteristics of the CT machine was demonstrated by the supplier following auto-performance test procedures using manufacturer supplied CT phantom/test tools. During acceptance measurements, CT phantoms were scanned and APT software calculated results were compared with the manufacturer specified limits. Such acceptance measurements provide performance results mainly related to image quality. Several mechanical parameters such as CT and pseudo center, orthogonality and flatness of CT couch with respect to imaging plane, etc, are not tested during APT. However, validation of these parameters is important from virtual simulation viewpoint. Even though CT dose is measured at the factory site, independent validation of the manufacturer quoted CTDI and system software calculated CTDI_w_ is important and recommended[5,6] from radiation safety point of view. Moreover, APT results need to be validated independently before it is used as baseline value for routine QA of the CT scanner. Thus there is a need for comprehensive evaluation of CT scanner during installation as recommended by AAPM.[5,6]

The moving laser system is calibrated selecting pseudo CT center as its origin. Accurate localization of CT center and pseudo CT center therefore ensures accurate positioning of isocenter on patient during virtual simulation procedure. The overall electromechanical performance of the CT scanner was found satisfactory as test results were within the specified tolerance limits.

CT number accuracy is clinically important as it is related to electron density of different tissues/organs, which is of paramount importance for obtaining accurate dose distribution. CT number accuracy and uniformity of water was well within the acceptable limit of 0 ± 2 HU in both APT and in independent measurement. However, CT number measured independently for other materials was higher than both theoretically calculated and nominal values quoted in the literature.[11] Independently measured CT number of air is equal to manufacturer quoted value of −1024 HU. However, it does not agree with the nominal and calculated values and is higher than the acceptable[12] value of −1000 ± 3 HU. CT number of air ranging from −960 to −994 HU has been reported in the literature[11] for CT scanners of different manufacturers. The wide range of CT number for air may be due to different contrast scale used in the calculation algorithm of different CT scanners. Though no standard CT number is available for other materials, nominal values quoted in the literature and calculated values for 60 keV X-rays were used for comparison. Garcia-Ramirez[10] *et al* has measured and compared CT number of air, water, LDPE (low density polyethylene), acrylic and teflon for CT scanners (AcQSim, UltraZ, GE High Speed) of different manufacturers. CT number of acrylic (120 ± 4 HU) was found very consistent for different CT scanners and the maximum variation of ± 4 HU was reported for GE HighSpeed. Our measured value (140 HU) is 20 HU higher than both calculated and nominal values (120 HU). Large deviation between measured and nominal CT number (990 HU) of teflon was reported for different CT scanners with a maximum deviation of 54 HU for GE High Speed. Our measured CT number (992 HU) of teflon is comparable to nominal value, whereas it is 22 HU more than the calculated value. Measured, calculated and nominal CT number of polypropylene is in agreement to one another within 10 HU. Nominal values for polystyrene, bakelite and nylon are not available for comparison. The difference in measured and calculated CT numbers of polystyrene, bakelite and nylon are 17, 94 and 46 HU respectively. While calculating CT number of different materials, we used the standard molecular formula and the principle of fractional weighting of the constituent elements to determine their linear attenuation coefficient. The compositional variation of these materials may be the reason for large difference in their calculated and measured values. The value of contrast scale (1.998 × 10^−4^ cm^−1^/CT number) derived from our measured CT numbers and linear attenuation coefficient of different materials is in excellent agreement to the recommended value of 2.0 × 10^−4^ cm^−1^/CT number.

Manufacturer specifies image noise as standard deviation of CT numbers of water at the center of the uniform water phantom, while it is most commonly expressed as percentage of μ_w_ and calculated using equation 2. Image noise value measured during APT for head type phantom is within the manufacturer specified limit of 13.7 ± 1.37 HU at 110 kVp and 12.9 ± 1.29 HU at 130 kVp. The σ value (13.58 HU at 110 kVp) measured during APT gives image noise as high as 1.32%. However, when similar water phantom was scanned in routine examination mode using the same scan parameters that were used during APT, the image noise calculated using average σ value (3.96 HU at 110 kVp) is 0.38%. The independently measured image noise for our CT scanner is comparable to the image noise range (0.3 - 0.35%) reported[11] for the different CT scanners. Difference in contrast scales employed during APT and during routine scan could be the reason for large difference in image noise of APT and of independent method.

CT dose is most commonly expressed in terms of CTDI and CTDI_w_.[5,6] Our measured and manufacturer specified CTDIs and CTDI_w_ for head and body phantoms are well within the manufacturer specified limit of ± 20%. However, to compare our data with similar CT scanners of other manufacturers, CTDIs on the surface and at the center of the phantom were measured at 120 kVp and 260 mAs using 1 cm slice thickness. Measured CTDIs on surface (3.6 cGy) and at center (3.3 cGy) of head phantom for our single slice Somatom Emotion CT scanner are comparable to reported[11,12] CTDI values (3.2 - 4.0 cGy on surface and 3.6 - 3.7 cGy at the center) of similar CT scanners of other manufacturers and are also within the industry quoted[11,12] CTDI range of 3.2 - 7.6 cGy for all scanners. Moreover, our measured CTDIs on surface (2.6 cGy) and at center (1.2 cGy) for body phantom are within the reported[11,12] CTDI values (2.1 - 2.6 cGy on the surface and 1.1 - 1.2 cGy at the center) of similar other scanners and are also within the industry quoted[11,12] CTDI range of 1.1-4.2 cGy for all scanners.

## Conclusions

Comprehensive QA of a CT scanner used for virtual simulation was carried out using manufacturer supplied and in-house fabricated CT phantoms/test tools. Test results of in-house fabricated CT phantoms/test tools were comparable to test results of manufacturer supplied phantoms. The in-house fabricated phantoms/test tools are cost-effective in comparison to commercially available phantoms and can be locally fabricated. Test results of the QA parameters for the CT scanner tested are within the specified limit for its use in virtual simulation procedure. However, test results of some of the QA parameters obtained by independent method are different from the test results obtained by APT. This observation demonstrates the need for independent validation of auto-performance test results. Though comprehensive QA is recommended periodically, APT can be used for quick periodic quality control of the CT scanner.
